# Community assembly during vegetation succession after metal mining is driven by multiple processes with temporal variation

**DOI:** 10.1002/ece3.8882

**Published:** 2022-04-29

**Authors:** Ting Li, Huaju Yang, Xinting Yang, Zhaolai Guo, Denggao Fu, Chang’e Liu, Shiyu Li, Ying Pan, Yonggui Zhao, Fang Xu, Yang Gao, Changqun Duan

**Affiliations:** ^1^ 12635 School of Ecology and Environmental Sciences Yunnan University Kunming China; ^2^ 12635 Yunnan Key Laboratory for Plateau Mountain Ecology and Restoration of Degraded Environments Yunnan University Kunming China; ^3^ YICI Municipal Garden Engineering Co. Ltd Kunming China

**Keywords:** CSR strategies, dispersal limitation, functional trait space, metal pollution, null models, stochastic process

## Abstract

The mechanisms governing community assembly is fundamental to ecological restoration and clarification of the assembly processes associated with severe disturbances (characterized by no biological legacy and serious environmental problems) is essential. However, a systematic understanding of community assembly in the context of severe anthropogenic disturbance remains lacking. Here, we explored community assembly processes after metal mining, which is considered to be a highly destructive activity to provide insight into the assembly rules associated with severe anthropogenic disturbance. Using a chronosequence approach, we selected vegetation patches representing different successional stages and collected data on eight plant functional traits from each stage. The traits were classified as establishment and regenerative traits. Based on these traits, null models were constructed to identify the processes driving assembly at various successional stages. Comparison of our observations with the null models indicated that establishment and regenerative traits converged in the primary stage of succession. As succession progressed, establishment traits shifted to neutral assembly, whereas regeneration traits alternately converged and diverged. The observed establishment traits were equal to expected values, whereas regenerative traits diverged significantly after more than 20 years of succession. Furthermore, the available Cr content was linked strongly to species' ecological strategies. In the initial stages of vegetation succession in an abandoned metal mine, the plant community was mainly affected by the available metal content and dispersal limitation. It was probably further affected by strong interspecific interaction after the environmental conditions had improved, and stochastic processes became dominant during the stage with a successional age of more than 20 years.

## Graphical abstract

Based on the functional traits of plants, the assembly rules of the early and middle stages of vegetation succession were revealed. It provides theoretical basis for restoration activities after serious disturbance.

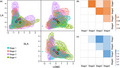

## 1 INTRODUCTION

Plant community assembly, a fundamental process in community ecology, is a critical factor for the prediction of ecosystem responses following disturbance (Purschke et al., 2013). For this reason, an understanding of community assembly rules can inform the protection, management, and restoration of disturbed ecosystems. Despite the publication of a considerable amount of literature on community assembly since 1916 (Clements & Edward, 1916), studies have yielded contradictory conclusions. Evidence for assembly mechanism has been provided by studies of succession in naturally regenerated communities following natural disturbances (natural communities), after which there are more or less biological legacy [defined as living organisms, perennating structures, dormant spores and seeds, organic debris, or any biologically derived material in soils (Ferreiro et al., 2018)], and not very harsh environmental conditions, such as old fields, degraded grasslands, and secondary forests (van Breugel et al., 2019; Helsen et al., 2013; Purschke et al., 2013); In contrast to the understanding achieved for natural communities, little is known about the assembly rules in communities following severe disturbance characterized by no biological legacy and serious environmental impacts, such as mining. It remains unclear whether these rules differ from those in natural communities. An understanding of the assembly rules specific to different situations can provide insight into successional mechanisms, particularly for severely disturbed ecosystems, and is important in ecological restoration (Wainwright et al., 2018).

Community assembly is driven by multiple deterministic and stochastic processes and controlled by biotic and abiotic factors. Without considering geographic scale, studies have demonstrated that propagule availability (i.e., dispersal limitation), abiotic environmental conditions (i.e., environmental filtering), and species interactions (i.e., limiting similarity) determine plant community assembly (Götzenberger et al., 2012). Researchers have recognized that colonization drives community assembly; thus, species with strong colonization abilities are expected to dominate during the early stages of succession (Caccianiga et al., 2006), whereas increased diversity during later stages may result in increased interspecific and intraspecific competition (Groninger et al., 2017); that is, community assembly is determined by biotic processes. However, studies have yielded the contradictory finding that community assembly is driven mainly by stochastic processes during the early stages of succession (Marteinsdóttir et al., 2018) and not by competition in the later stages (Li et al., 2015). Adverse environmental conditions can result from severe anthropogenic disturbances (Miles & Walton, 1993; Walker & Del Moral, 2003); and mining is among the severe disturbances that are associated with serious environmental problems. Thus, environmental filtering may be the dominant process in community assembly in early succession of abandoned metal mines; as conditions improve, the limitations imposed by colonization and competition may play more important roles in late succession.

Drivers of community assembly during plant succession are identified based on community responses, which are in turn typically based on composition [e.g., cover (Durbecq et al., 2020), richness (Purschke et al., 2013), phylogeny (Xu et al., 2017), and functional diversity (FD) (Purschke et al., 2013, 2017)]. Functional traits are key indicators of plant community assembly (Meiners et al., 2015) and clearly reflect causal, organism–organism, and organism–environment relationships (Gillison, 2013). Functional traits can accurately predict ecosystem responses to disturbance (Purschke et al., 2013). Because individual traits do not sufficiently reflect community strategies and integrated functions (Loranger, Blonder, et al., 2016; Mao et al., 2018), the mechanisms of co‐occurrence in communities are typically inferred based on comprehensive functional traits (combination of functional traits) (Kraft et al., 2008). Ecological strategies reflect the comprehensive traits of individuals or communities (Büchi & Vuilleumier, 2016; Rosado & de Mattos, 2017) based on the CSR classification proposed by Grime (Grime, 1977), in which C (competitiveness) is selected in low‐pressure disturbed environments (Grime, 1977), S (stress tolerance) is characterized by slow growth and resource allocation, with the aim of resisting stress (Gillison, 2013), and R (rurality) is characterized by high productivity and seed output and is best suited to low‐stress, high‐disturbance environments (Grime, 1977). These traits may result from interactions between species and their environments—that is, environmental filtering, in which plants adapt to environmental conditions by adjusting their functions (Boukili & Chazdon, 2017; Tiselius et al., 2019). Comprehensive functional traits also may be determined based on propagule availability during the early and late stages of succession (Makoto & Wilson, 2019). Moreover, competitive exclusion (Abrams, 1983) precludes the co‐existence of species that share the same traits (Kunstler et al., 2016).

Metal mining exerts long‐lasting effects and is considered a severe disturbance (Prach et al., 2019) owing to its significant detrimental effects on ecosystems (Chaturvedi & Singh, 2017). Numerous abandoned mines are found throughout the world; these reduce land use, cause soil erosion, and act as sources of heavy metal contamination (Wang et al., 2013). Soil of post‐mining sites had high concentrations of heavy metals (Grigholm et al., 2016; Punshon et al., 2016) and deficient nutrients (Groninger et al., 2017). These sites are typically used for ecological restoration, with human assistance, based on the principles of ecological succession. As recent evidence suggests that spontaneous succession can yield satisfactory target vegetation communities (Li et al., 2022), it is crucial to identify barriers to this process for optimal restoration decision‐making (Aradottir & Halldorsson, 2018). Hence, we analyzed data collected from Lao Chang, an abandoned metal mine in southwestern China, to identify the mechanisms of community assembly after mining and variations in these drivers with successional age.

We used a chronosequence approach in which vegetation patches of different ages represented communities at different successional stages (Purschke et al., 2013). By comparing the comprehensive traits of communities at different stages, we identified the key mechanisms and environmental factors driving plant community assembly and succession following mining. We hypothesize that with the successional age, the diversity of plant community and function increases and the environmental conditions improved. Community assembly is significantly affected by environmental filtering in the early stage of succession. We expect to explore the assembly mechanism of plant community in different succession stages based on these.

## 2 MATERIALS AND METHODS

### 2.1 Study area

The study was conducted at Lao Chang (103°10′51.96″E, 23°17′26.95″N, 2437 m a.s.l.), an abandoned metal mine surrounded by *Pinus yunnanensis* forests in Gejiu, Yunnan Province, China (Figure S1). Open‐pit mining was conducted at Lao Chang for more than a century, followed by mixed‐tunnel mining in the mid‐20th century. Operations ceased gradually beginning in the 1990s, but no measure was taken to promote restoration.

Four sites were selected to represent different successional stages based on the time of abandonment: (1) a site in the primary stage of succession, where mining ceased around 2017, representing 2–3 years of succession and sparsely vegetated by grasses (Stage 1); (2) an early successional site abandoned around 2014, representing approximately 5 years of succession and dominated by a high grass (Stage 2) community; (3) an early‐mid‐successional site abandoned around 2004, representing 15 years of succession and dominated by shrubs (Stage 3); and (4) a mid‐successional site abandoned in the late 1990s, representing more than 20 years of succession and dominated by scrub grassland with sparse trees (Stage 4).

### 2.2 Vegetation and soil sampling

In late November 2019, near the end of the rainy season, we randomly established three plots with three spatial scales at each site (Tardif et al., 2019) (Figure S1). First‐order plots were square plots, each with areas of 100 m^2^ (10 × 10 m), in which the abundance of all woody species was recorded. The second‐order plots were circular plots with a radius of 1.78 m and area of 10 m^2^, and the remaining third‐order plots were square plots with areas of 1 m^2^ (1 × 1 m). Abundance of all shrubs in the second‐order plots and all herbaceous species in the third‐order plots were recorded, respectively.

Three 0–10‐cm‐depth soil samples were collected from each site, and five soil properties were measured: total metals, available metals (that can be absorbed by plants, like water‐soluble state, exchangeable state, etc.), physical properties, ionic properties, and nutrients. Total Cr, Cd, Cu, Ni, Pb, and Zn, and available Cr, Cd, Cu, and Pb contents were obtained by digesting soil samples in an HCl‐HNO_3_‐HF‐HClO_4_ system and extracting in a DTPA‐TEA‐Ca(NO_3_)_2_ system, respectively, and determined by flame atomic absorption spectrometry using an Agilent AA240 spectrometer (Agilent Technologies, Santa Clara, CA, USA). The total Sn was directly determined in situ using the portable X‐ray fluorescence analyzer (XLt 794; Niton, Winchester, UK). The physical properties measured included the in situ soil temperature and moisture content (MC). Ionic properties included the oxidation–reduction potential (ORP), pH (1:5 soil: water ratio), and electrical conductivity (EC). The total potassium content was measured using atomic absorption spectrometry, and the total phosphorus content was measured using spectrophotometry. The total nitrogen and carbon were directly measured using a TOC analyzer (Vario TOC cube, Elementar, Hanau, Germany).

### 2.3 Functional traits

We collected data on eight functional traits related to establishment and regeneration (Table 1). The establishment traits included three key leaf traits (Dayrell et al., 2018): the leaf area (LA), which represents energy and water balance (Diaz et al., 2016) and photosynthesis (Dayrell et al., 2018), and the specific leaf area (SLA) and leaf dry matter content (LDMC), which represent resource acquisition capacity, water use, life span, and stress tolerance (Dayrell et al., 2018; Diaz et al., 2016; Gillison, 2013; Gratani & Bombelli, 2000). These three traits (Ghnaya et al., 2015; Wu et al., 2020) indicate adjustment and adaptation to stress. We sampled at each site, and leaf traits were measured on six individuals per species with abundance ≥5 in each plot. The LA was quantified by scanning the leaf samples using an LA meter (CI‐203; CID Bio‐Science, Camas, WA, USA). The SLA was determined by dividing the LA by the leaf dry weight, and the LDMC was obtained by dividing the dry leaf weight by the fresh weight. The dry weight was assessed after oven drying at 70°C to constant weight, and the fresh and dry weights were measured using an electronic balance with a precision of 0.0001 g. The regenerative traits examined included the seed mass, lengths of the flowering and fruiting periods, first month of flowering (January–December = 1–12, respectively; year‐round = 13, clonal = 0), and longevity. Based on life span and reproductive mode, longevity was classified as (1) annual and biological, (2) annual to perennial, (3) perennial and non‐clonal, and (4) perennial and clonal (Marteinsdóttir et al., 2018). Seed mass data were obtained from the Seed Information Database, and other regenerative trait data were obtained from the Scientific Database of Chinese Plant Species. Missing data, which comprised 14.3% of all data, were estimated using the “rpart (ver. 4.1‐15)” package in R (ver. 4.0.4).

**TABLE 1:** List of plant functional traits

Type	Trait	Abbreviation	Unit	Description
Establishment traits	Specific leaf area	SLA	mm^2^/g	Leaf area divided by leaf dry weight
	Single leaf area	LA	mm^2^	Obtained using a leaf area meter
	Leaf dry matter content	LDMC	–	Leaf dry weight divided by fresh weight
Regenerative traits	Seed mass	SM	g	Average mass of 1000 seeds
	Longevity	–	Ordinal classes	(1) Annual and biological, (2) annual to perennial, (3) perennial and non‐clonal, and (4) perennial and clonal
	Length of flowering period	Flowering period	Months	–
	Length of fruiting period	Fruiting period	Months	–
	First month of flowering	FMF	Ordinal classes	January–December = 1–12; year‐round = 13, clonal = 0

### 2.4 Comprehensive traits and null models

At present, null models based on species traits are commonly used to explore the mechanisms of community assembly (Hardy, 2008; Marteinsdóttir et al., 2018; Purschke et al., 2013). We randomly sampled the species pool (all species recorded in each plot) to construct simulated communities and inferred the dominant drivers of community assembly by comparing simulated and observed community traits.

To explore the mechanisms of community assembly at different successional stages, we calculated the functional trait space (FTS) and mean pairwise functional distance (MFD), the FTS requires the data to be numeric values, and MFD is used when the data are categorical type and constructed two null models for each stage to determine the dominant drivers of succession by comparing observed and expected values. The larger observation value statistically expressed as trait divergence indicates that the assembly may be driven by competition, whereas trait convergence is most likely driven by environmental filtering or dispersal limitation. Similarity of observed and expected values was taken to indicate the dominance of stochastic processes (Götzenberger et al., 2012; Marteinsdóttir et al., 2018). Trait convergence and divergence were used to infer the dominant processes of community assembly and to identify the mechanisms driving succession at different stages (Meiners et al., 2015).

The FTS for the comprehensive leaf traits was used to represent establishment (Benavides et al., 2019). The n‐dimensional hypervolume proposed by Hutchinson is used widely in ecology (Cooke et al., 2019; Jarvis et al., 2019; Pigliucci, 2007), particularly in FTS construction (Lamanna et al., 2014; Loranger, Violle, et al., 2016) and is calculated from data in n‐dimensional space. The geometric parameters of the hypervolume may be expressed by statistics (Blonder et al., 2014) representing the variation (volume), comprehensive trait value (centroid) (Benavides et al., 2019), and similarity (overlap, minimum and maximum distances) between two functional trait datasets (Mammola, 2019). A three‐dimensional hypervolume based on the LA, SLA, and LDMC was constructed to quantify the FTS at each successional stage (Hutchinson, 1957). Based on the hypervolume calculation method presented by (Blonder et al., 2014), we standardized the observed leaf trait values and used the support vector machine method to maintain correlations among trait axes (Cooke et al., 2019). Simulated values were sampled from the observed values for each successional stage and used to estimate FTS overlap and centroid distance, which reflect differences among successional stages (Mammola, 2019). Analyses were performed using the “hypervolume” (ver. 2.0.12) package (Blonder et al., 2017) in R (ver. 4.0.4).

The MFD is used widely in community ecology and can accommodate missing data and categorical variables (Li et al., 2015; Purschke et al., 2013). Because our regenerative traits included categorical variables, we used MFDs to characterize different communities’ regenerative traits (Marteinsdóttir et al., 2018). The distance matrix was calculated using the Gower distance in the “Picante” package (ver. 1.8.2) (Kembel et al., 2010) and then associated with species distribution; MFD is calculated by “mpd” function.

For each successional stage, we simulated 999 FTSs and MFDs using the null models and compared these with observed values based on a permutation test using the “as.randtest” function of the package “ade4” (ver. 1.7‐17) (Dray & Dufour, 2007). We calculated the effect size (ES) value [ES = 2*(*P*–0.5)], which is the statistic of the permutation test and ranges from –1 to 1, where *P* is the sum of the probability that the simulated value is less than the observed value and half the probability that they are equal. A negative ES indicates that observed values are markedly smaller than simulated values and vice versa, whereas an ES close to zero indicates no difference between observed and simulated values (Bernard‐Verdier et al., 2012). For FTS, we randomly sampled from the pool of observed establishment traits in all succession stages. Sample size varied according to the number of observed values (Bernard‐Verdier et al., 2012; Loranger, Violle, et al., 2016). The convergence of establishment traits indicates that community assembly may be shaped by environmental filtering (Götzenberger et al., 2012; Marteinsdóttir et al., 2018). For the MFD, a random taxonomic matrix was constructed by randomly sampling from the pool of all observed species; the sample size was equal to the species richness of each community. The convergence of regenerative traits very likely indicates that dispersal limitation dominates community assembly (Götzenberger et al., 2012; Marteinsdóttir et al., 2018). For the establishment and regenerative traits, divergence indicates that community assembly might be driven by competition or environmental heterogeneity (Funk et al., 2017), whereas stochastic processes are possible to dominate when the observed and expected values are equal (Götzenberger et al., 2012).

### 2.5 CSR strategy scores

We calculated an ecological strategy score for each site to clarify the key environmental factors influencing community assembly. Relative to the taxonomic composition, trait composition (i.e., ecological strategy) provides a better reflection of community assembly processes (Helsen et al., 2013). CSR strategies can be estimated based on three key leaf traits (Pierce et al., 2017). We calculated a CSR score and CSR strategy for each individual using the “StrateFy” calibration tool (Pierce et al., 2017) based on the LA, SLA, and LDMC, which all correlated significantly with CSR strategies. CSR strategy score distributions were plotted using the “ggtern” (ver. 3.3.5) R package.

### 2.6 Statistical analysis

To understand the differences in plant diversity and composition at different succession stages, we calculated the Shannon–Wiener index (*H*'), species richness, and Whittaker index (*βw*) of plant communities. The differences between succession stages were analyzed via Kruskal–Wallis test and Dunn test conducted by “kruskal.test” function and “dunnTest” function from “FAS” (ver. 0.9.3.) package (Ogle, 2018). ANOVA and the LSD tests from “agricolae” (ver. 1.3‐5) package (de Mendiburu & de Mendiburu, 2019) were used to explore the differences in soil properties among successional stages. Prior to this, all soil factor data were log‐transformed. Shapiro test and Bartlett test in R (ver. 4.0.4) were used to test the normality and homogeneity of data, respectively.

To clarify the changes in functional patterns of plant communities during succession, the differences among the various successional stages in establishment traits were evaluated using Kruskal–Wallis test and the Dunn test. Since regeneration traits are categorical variables, we calculated the FD of each regeneration trait. FD is expressed as the mean pairwise distance between species within each community (Petchey & Gaston, 2002). Firstly, calculate the Gower's distance of functional traits, which is for categorical traits, and then calculate the mean pairwise distance according to species abundance, richness, and present‐absence for per plot (Petchey & Gaston, 2002); this was calculated using the “philentropy” (ver. 0.5.0) R package (Drost, 2018). The differences of FD in regeneration traits among successional stages were calculated via ANOVA and the LSD tests. In order to clarify the differences of MFD in different succession stages, ANOVA and LDS tests were used.

In order to explore the environmental factors affecting ecological strategies, we used a Mantel test to analyze correlations between soil properties and community ecological strategies. Beta regression models were also generated, including factors significantly correlated with ecological strategies. The Mantel test was performed using the “vegan” R package (ver. 2.5‐7) (Oksanen et al., 2013); soil properties were transformed into a dissimilarity matrix a priori (Figures S4 and S5). Beta regression was conducted using the “betareg” package (ver. 3.1‐4) (Cribari‐Neto & Zeileis, 2010) and plotted using the “ggplot2” (ver. 3.3.5) R package (Wickham et al., 2016).

## 3 RESULTS

### 3.1 Soil properties and vegetation community in successional stages

**FIGURE 1:**
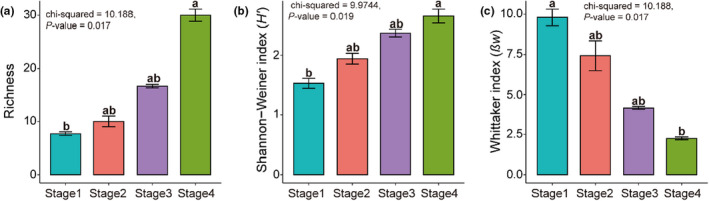
Taxonomic diversity of plant communities between successional stages (*n* = 12 plots). (a) Species richness, (b) Shannon–Wiener index (*H’*), and (c) Whittaker index (*βw*) (mean ± *SE*) of the vegetation community at different successional stages. Different letters indicate significant differences between stages based on 95% confidence intervals (*p* < .05) obtained using Kruskal–Wallis test and Dunn test. Stage 1 = 2–3 years, Stage 2 = 5–6 years, Stage 3 = 15 years, and Stage 4 > 20 years

Species richness and the Shannon–Wiener index (*H*') increased with successional stage; by contrast, Whittaker's index (*βw*) decreased with successional stage (Figure 1).

Total Cu decreased significantly to 643.75 mg/kg during Stage 3, and Total Sn decreased to 643.333 mg/kg during Stage 4. Available Cr and available Cu in Stage 1 were significantly higher than during other stages, at 0.415 mg/kg and 44.405 mg/kg, respectively. The ORP and MC observably increased during Stage 3, reaching 292.333 mv and 28.222%, respectively. In terms of nutrients, Stage 3 had significantly higher TN, and the carbon‐nitrogen (C: N) ratio in Stage 3 was significantly higher than that in Stage 1 (Table S1).

### 3.2 Changes in vegetation traits over time

**FIGURE 2:**
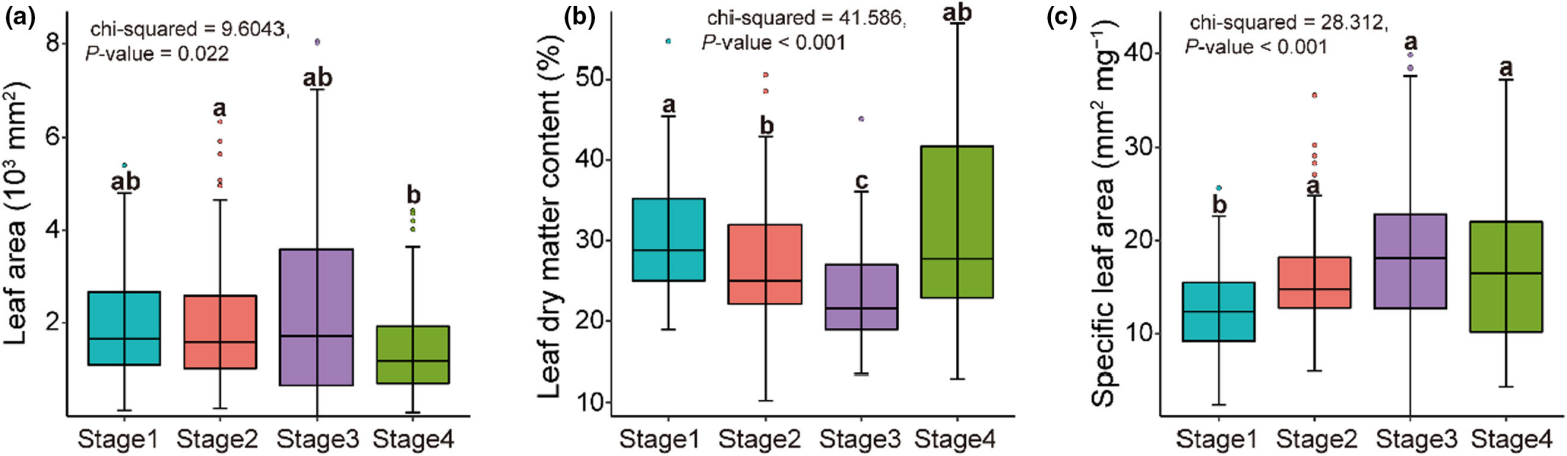
Leaf traits of plant communities between successional stages (*n* = 309 plant individuals). (a) Leaf area, (b) leaf dry matter content, and (c) specific leaf area of the vegetation communities at different successional stages. Different letters indicate significant differences between different stages based on 95% confidence intervals (*p* < .05) obtained using Kruskal–Wallis test and Dunn test. Stage 1 = 2–3 years, Stage 2 = 5–6 years, Stage 3 = 15 years, and Stage 4 > 20 years

**FIGURE 3:**
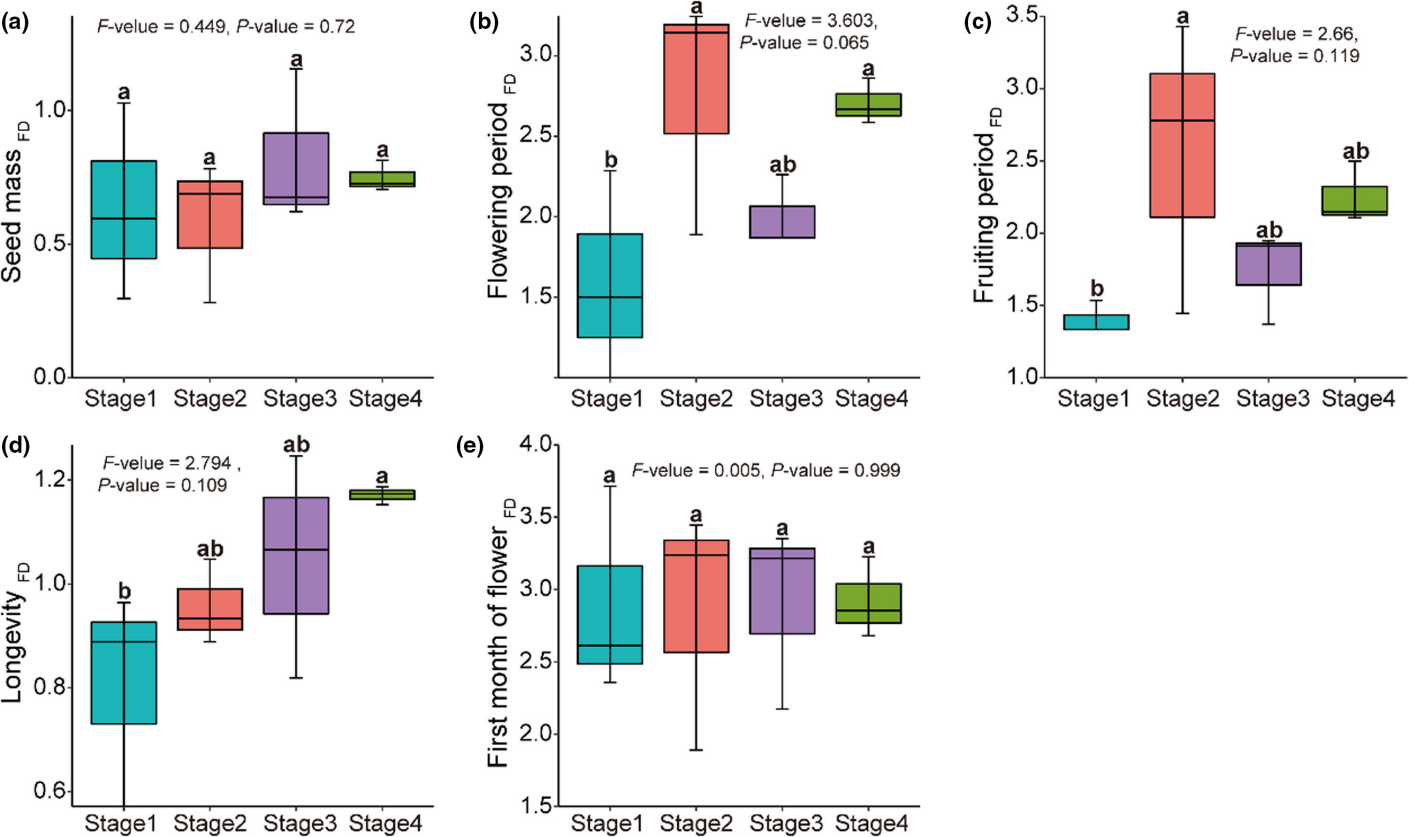
Functional diversity of five regenerative traits in different successional stages (*n* = 12 plots) (a) seed mass, (b) flowering period (c) fruiting period, (d) plant longevity, and (e) first month of flowering. Functional diversity is expressed as the mean pairwise distance between species within a community. Different letters indicate significant differences between stages based on 95% confidence intervals (*p* < .05) obtained using ANOVA and the LSD tests. Stage 1 = 2–3 years, Stage 2 = 5–6 years, Stage 3 = 15 years, and Stage 4 >20 years

Regarding establishment traits, leaf dry matter content was lowest during Stage 3, and the specific leaf area was lowest during Stage 1 (Figure 2). Regarding regeneration traits, the flowering and fruiting periods during Stage 1 were shorter than those during Stage 2, and plant longevity was significantly lower during Stage 1 than Stage 4 (Figure 3).

**FIGURE 4:**
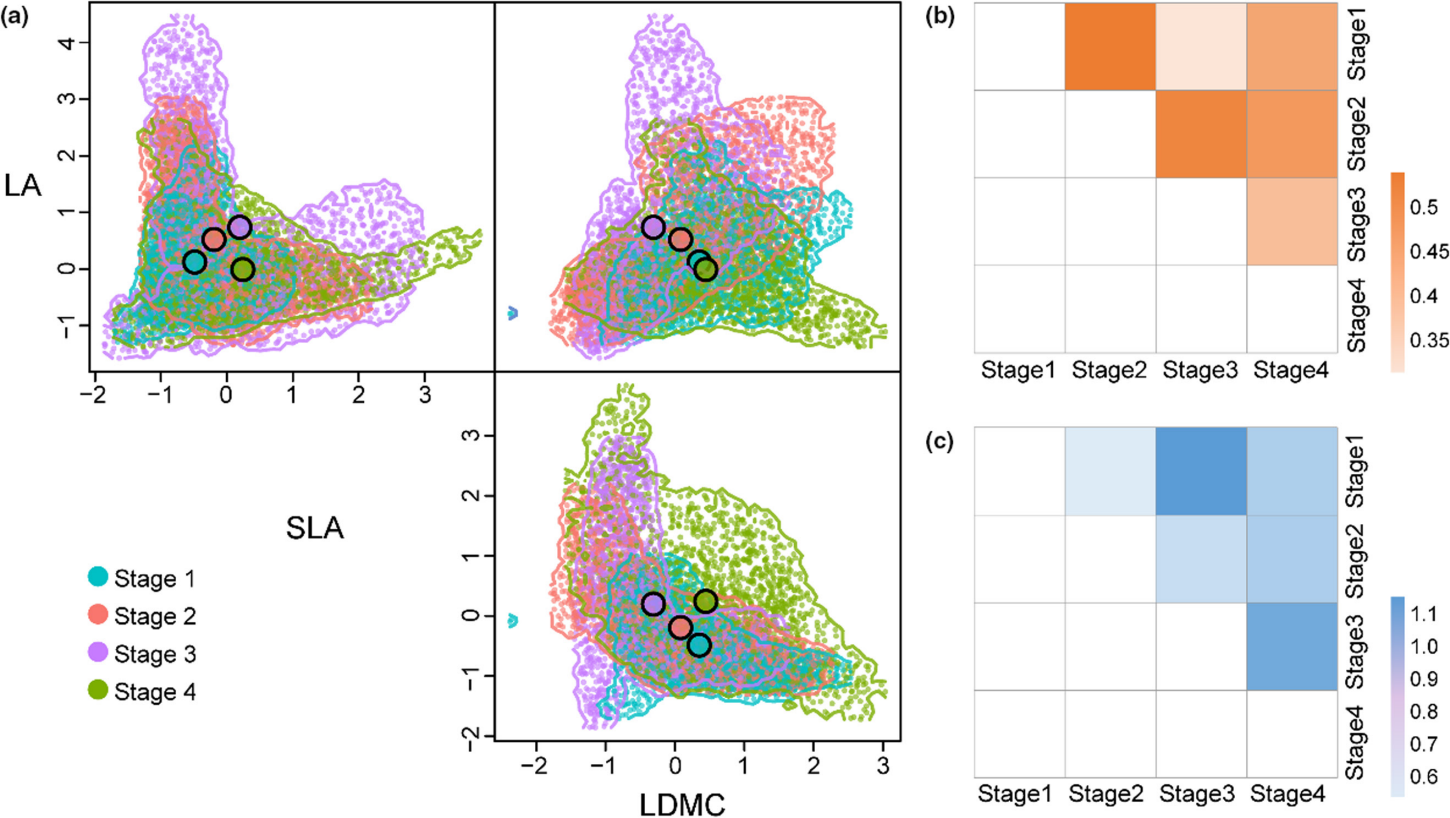
Functional trait space (FTS) of three establishment traits among four successional stages (*n* = 12 plots), shown as (a) three functional trait axes: LA, SLA, and LDMC. The larger points represent the centroid of the hypervolume, and the smaller solid and transparent points represent observed and simulated values, respectively; (b) hypervolume overlap, based on the Jaccard similarity between two hypervolumes calculated by the volume of intersection of 1 and 2 divided by the volume of union of 1 and 2; and (c) centroid distance, obtained based on the Euclidean distance between the centroids of two hypervolumes at different stages of succession, where color represents the size of the value; also, the more overlap and smaller the centroid distance, the smaller the difference in comprehensive traits between the two stages and vice versa. LA = single leaf area, SLA = specific leaf area, LDMC = leaf dry matter content. Stage 1 = 2–3 years, Stage 2 = 5–6 years, Stage 3 = 15 years, and Stage 4 > 20 years

The comparison of observed traits and null model results revealed that establishment traits shifted from lower than expected to expected values as succession progressed, whereas regenerative traits shifted from convergence to divergence in the first two successional stages, and repeated this pattern in the latter two stages (Table 2). With respect to the FTS of establishment traits, we observed greater hypervolume overlap and shorter centroid distances between Stage 2 and Stage 1 and between Stage 2 and Stage 3, whereas Stage 1 and Stage 3 exhibited opposite trends (Figure 4). The MFD for regenerative traits did not differ significantly with successional age (Figure S3).

**TABLE 2:** Effect size (ES) of each successional stage based on comparison of functional traits with null models

	Stage 1	Stage 2	Stage 3	Stage 4
ES_establishment traits_	−1	−0.980	−0.766	−0.026
ES_regenerative traits_	−1	0.998	−0.996	0.998

Note

A negative ES indicates that the observed value is strikingly smaller than the simulated values and vice versa; no difference was indicated by an ES close to 0. Stage 1 = 2–3 years, Stage 2 = 5–6 years, Stage 3 = 15 years, and Stage 4 > 20 years.

In Stage 1, the convergence of establishment traits (ES_establishment traits_ = –1) and regenerative traits (ES_regenerative traits_ = –1). In Stage 2, the establishment traits converged (ES_establishment traits_ = –0.980), whereas the regenerative traits diverged (ES_regenerative traits_ = 0.998). After more than 20 years of succession (Stage 4), no significant difference between expected and observed values was observed for the establishment traits (ES_establishment traits_ = –0.026); however, the regenerative traits diverged significantly (ES_regenerative traits_ = 0.998) (Table 2).

### 3.3 Key environmental drivers

**FIGURE 5:**
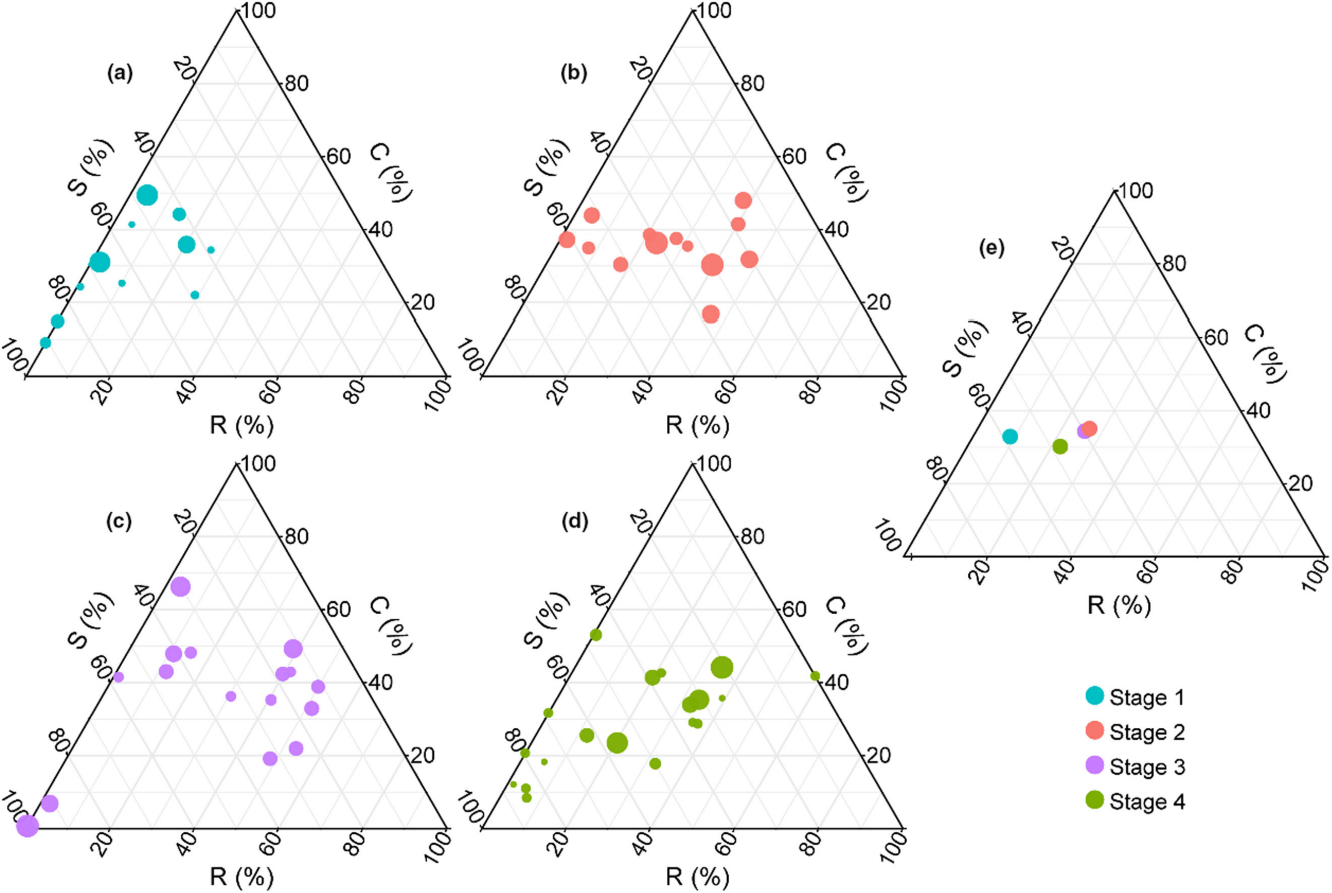
CSR strategy scores of each species at different succession stages and the average scores. C (competitiveness) is selected in low‐pressure disturbed environments, S (stress tolerance) is characterized by slow growth and resource allocation to resist stress, and R (ruderality) is characterized by high productivity and seed output and is best suited to low‐stress, high‐disturbance environments. The axes of the ternary plot represent the scores of the CSR strategies in (a) Stage 1 (*n* = 12 species), (b) Stage 2 (*n* = 13 species), (c) Stage 3 (*n* = 17 species), and (d) Stage 4 (*n* = 19 species), and (e) shows the average score for each succession stage. The point size in (a)–(d) indicates the relative species abundance. Stage 1 = 2–3 years, Stage 2 = 5–6 years, Stage 3 = 15 years, and Stage 4 > 20 years

**FIGURE 6:**
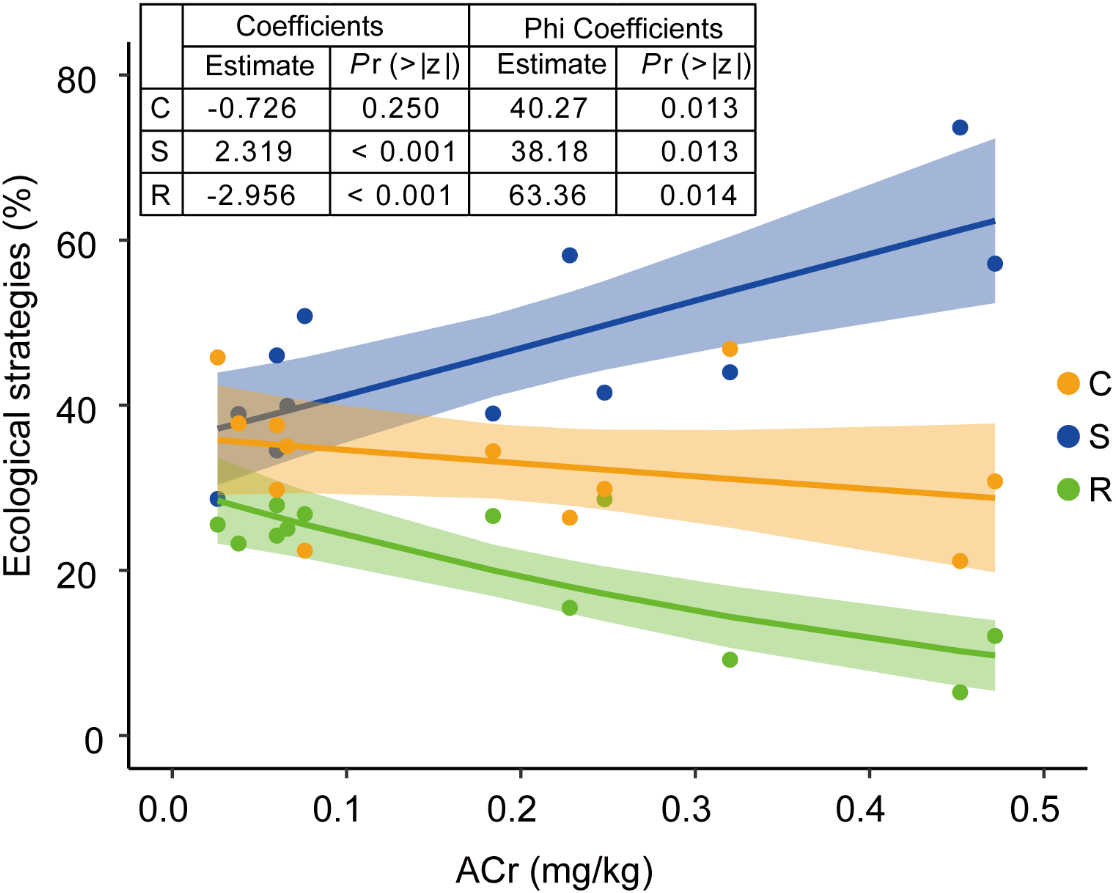
Relationship between mean ecological strategy scores of the plant communities and available Cr (ACr) content. Estimate and *p*‐values are obtained by beta regression analysis (*n* = 12 plots). C, S, and R represent competitor, stress‐tolerant, and ruderal, respectively, and C + S + R = 100%. The shading represents the 95% confidence intervals. Dots are individual observations

In Stage 1, a S/CSR strategy was established with a larger S component, while Stages 2 and 3 used a CSR strategy without a dominant component among C, S, and R (Figure 5). The strategies correlated significantly with available Cr (*p*_adjust_ = .032; Table 3). The available Cr corresponded with the increased importance of the S strategy (*p* < .001) and decreased importance of the R strategy (*p* < .001; Figure 6).

**TABLE 3:** Mantel test between the soil property difference and vegetation‐strategy composition^a^

Soil properties	*r*	*p*_adjusted_
Total Cr	.351161	.16
Total Zn	−.1701	.949
Available Pb	−.10952	.932
Available Cr	.545532^*^	.032
Temperature	−.01529	.6992
pH	−.23299	.949
Total K	.07081	.556
Total P	.206533	.288

Note

*r* is the correlation coefficient, and a *p*‐value < .05 is considered significant.

^a^ Vegetation ecological strategy refers to the comprehensive traits of vegetation based on the CSR classification, where C (competitiveness) is selected in low‐pressure disturbed environments, S (stress tolerance) is characterized by slow growth and resource allocation to resist stress, and R (ruderality) is characterized by high productivity and seed output and is best suited to low‐stress, high‐disturbance environments.

## 4 DISCUSSION

### 4.1 Environmental filtering

The convergence of establishment traits might indicate that initial community assembly might be controlled strongly by environmental conditions and that the influence of environmental filtering decreased gradually as succession progressed. Colonization is generally considered to be the dominant process driving the succession of natural communities (Li et al., 2015). In addition, some studies have revealed the importance of environmental filtering in community assembly (Conradi et al., 2017; Méndez‐Toribio et al., 2020; Purschke et al., 2017; van Breugel et al., 2019). These trends are in accordance with our observations of succession in the context of severe anthropogenic disturbance. Environmental filtering was clearly among the key mechanisms driving species assembly, which may be attributable to environmental and geological problems caused by anthropogenic activities. The wastelands created by metal mining are characterized by high concentrations of heavy metals (Grigholm et al., 2016; Punshon et al., 2016) and low soil fertility (Groninger et al., 2017); as such, changes in community assembly can be explained by changes in environmental conditions (Mao et al., 2018). In harsh environments, community functional traits are limited by environmental conditions (e.g., water, fertility, and heavy metal concentrations) (Raevel et al., 2012; Vitti et al., 2020), resulting in functional convergence. However, succession is a dynamic process, and these conditions improve over time (Kapusta & Sobczyk, 2015; Wang et al., 2018). As succession proceeds from bare land to plant communities with high FD, the status and content of heavy metals change, the pH shifts, and the nutrient content and water holding capacity of the soil increase (Nikolic et al., 2016; Tardif et al., 2019). Thus, the early stages of succession may be favoring stress tolerators; its influence declines as succession progresses.

After more than 20 years of succession, stochastic processes were possible to dominate community assembly. Thus, during the mid‐succession stage, community assembly may be controlled by stochastic factors, such as the seed emergence rate, seed rain events (Marteinsdóttir et al., 2010), and random colonization (Ulrich et al., 2016). This finding contradicts evidence from previous studies, which suggests that stochastic processes are strong drivers in the early stages of succession (Chai et al., 2019; Marteinsdóttir et al., 2018). This contradiction may be attributable to the severity of the anthropogenic disturbance and heavy metal pollution at the metal mine, which created an environment hostile to the colonization and growth of most species; it took time for the hostile environmental conditions to abate sufficiently for stochastic processes to dominate, as is expected during early succession in more benign conditions.

In our study area, the available Cr concentration was the key factor influencing community functional traits. The literature indicates that changes in environmental conditions influence trait diversity (Mason et al., 2012); because environmental conditions differ among ecosystems, key influencing factors also differ. For example, community assembly in tropical dry forests is water‐limited (Méndez‐Toribio et al., 2020), whereas abundance in tropical forests correlates positively with soil nutrients (van Breugel et al., 2019). Similarly, community assembly in successional grasslands is driven by soil nutrients (Conradi et al., 2017), whereas the key drivers in subtropical forests are soil properties and light (Purschke et al., 2017). Soil carbon was found to drive the functional composition of a glacial outwash plain (Marteinsdóttir et al., 2018). The above researches showed that the key influencing factors should be related to the site conditions, a distinctive characteristic of abandoned metal mines is the soil's high metal content (Pajak et al., 2018), so the heavy metals in our site are likely to be the limiting factors of community assembly. Through absorption, transformation, and stabilization by plants (Williams et al., 1977) as well as a series of geochemical processes, heavy metal concentrations and toxicity are reduced over time (Chen et al., 2019). Thus, plants use more resources for detoxification and allocate fewer resources to growth and reproduction (Grime, 1977; Williams et al., 1977). Studies have demonstrated that excessive heavy metal pollution can affect future plant performance (e.g., cause low pollen viability and persistent metal resistance) (Chmielowska‐Bąk & Deckert, 2021); thus, metal content is a long‐term limiting factor.

### 4.2 Alternating dominance of dispersal limitation and interspecific interactions

The regenerative trait patterns in this study indicated that dispersal limitation and competitive exclusion likely dominated community assembly as succession progressed alternately. These results are consistent with those of other studies, which have shown that pioneer species have good colonization ability (Caccianiga et al., 2006) and that community assembly in most ecosystems is driven initially by dispersal limitation (Makoto & Wilson, 2019) and later by competition (Buma et al., 2019). Our results indicate that during succession following severe disturbance drivers of community assembly shift from dispersal limitation to competitive exclusion over a short time frame and that succession is driven by the alternation of these two processes.

Succession is a dynamic process in which plant communities continually alter soil conditions, facilitating the establishment of successive communities. Given the lack of soil seedbanks and nearby seed sources in the early stages of succession, pioneer species are required to allocate more resources to growth and reproduction and have short life spans (Grime, 1977). At this point, dispersal limitation is possible to dominate community assembly. As succession progresses, the availability of resources and seeds increases, and species are more likely to disperse and colonize (Chai et al., 2016). These processes promote increased diversity and thus increased competition, which is conducive to vegetation restoration and increased ecosystem function at abandoned metal mines (Zuppinger‐Dingley et al., 2014). At this stage, community assembly is likely driven by competitive exclusion. The early successional plant community alters environmental conditions through soil‐plant feedback and creates suitable conditions for later successional species, but in the process renders the environment less conducive to its own persistence (van de Voorde et al., 2011). Changes in environmental conditions provide opportunities for the introduction of new species, as the species present in the early stages of succession are stress tolerators with relatively poor competitive and reproductive capacities (Büchi & Vuilleumier, 2016), and as such are inhibited in the new environment (van de Voorde et al., 2011). At this point, it is reasonable to assume that during the first stage, new colonists lack seed sources, and in the following stage, as species abundance and FD increase, where competitive exclusion is possible the dominant process in community assembly. Another explanation offered by recent studies is that environmental limitations may cause the divergence of traits. Because a hostile environment abates at an uneven rate during the plant succession process, environmental heterogeneity may lead to different species strategies (Funk et al., 2017).

## 5 CONCLUSION

During succession in the context of severe anthropogenic disturbance, community assembly is driven by multiple mechanisms, and the importance of these processes changes as succession progresses. In the early stages of succession, community assembly was most likely limited by the available Cr content and dispersal limitation; as succession progresses, environmental filtering tends to weaken, and competitive exclusion may dominate after the initial stage. When environmental filtering is not the dominant process, assembly may be driven by stochastic processes. Hostile environmental conditions (e.g., heavy metal contamination) significantly influence community assembly.

## CONFLICT OF INTEREST

The authors declare no conflict of interest.

## AUTHOR CONTRIBUTIONS

**Ting Li:** Conceptualization (lead); Data curation (lead); Formal analysis (lead); Methodology (lead); Validation (lead); Writing – original draft (lead). **Huaju Yang:** Data curation (equal); Investigation (equal). **Xinting Yang:** Data curation (equal); Investigation (equal). **Zhaolai Guo:** Data curation (equal); Investigation (equal). **Denggao Fu:** Methodology (equal); Writing – review & editing (equal). **Chang'e Liu:** Supervision (equal); Writing – review & editing (equal). **Shiyu Li:** Supervision (equal). **Ying Pan:** Supervision (supporting); Writing – review & editing (supporting). **Yonggui Zhao:** Supervision (equal). **Fang Xu:** Resources (equal). **Yang Gao:** Resources (equal). **Changqun Duan:** Conceptualization (supporting); Funding acquisition (lead); Project administration (lead); Supervision (lead); Writing – review & editing (supporting).

## Supporting information

Supplementary Material

## Data Availability

Part of trait data are available from Seed Information Database (SID) (see http://data.kew.org/sid) and Scientific Database of China Plant Species (DCP) (see http://db.kib.ac.cn), and the other data available on request from the corresponding author.
