# Supplementary material for: Community assembly during vegetation succession after metal mining is driven by multiple processes with temporal variation
Source: Ecol Evol. 2022 Apr 29;12(5):e8882. doi: 10.1002/ece3.8882 (PMC9055294; doi:10.1002/ece3.8882)
Supplement: Supplementary file 1 — Supplementary Material [file ECE3-12-e8882-s001.docx]

**SUPPORTING INFORMATION**

***Community assembly during vegetation succession after metal mining is driven by multiple processes with temporal variation***

Table S1 Soil properties at different succession stages (mean ± SE, *n* = 12 plots). Different letters indicate significant differences between stages based on 95% confidence intervals (*P* < 0.05) obtained by ANOVA and the LSD. test. All data were log-converted before statistical analysis. Stage 1 = 2-3 years, Stage 2 = 5-6 years, Stage 3 = 15 years, Stage 4 > 20 years.

|  | | Successional stage | | | | *F*- value | *P*- value (> *F*) |
| --- | --- | --- | --- | --- | --- | --- | --- |
|  |  | Stage 1 | Stage 2 | Stage 3 | Stage 4 |  |  |
| Total metal | Total Cr (mg·kg^-1^) | 38.65 ± 4.02 b | 46.03 ± 3.49 ab | 58.40 ± 4.25 a | 56.23 ± 6.15 a | 2.948 | 0.0984 |
|  | Total Cd (mg·kg^-1^) | 66.76 ± 9.23 b | 86.80 ± 27.01 b | 196.11 ± 15.58 a | 105.78 ± 8.24 ab | 4.818 | 0.0335 |
|  | Total Cu (mg·kg^-1^) | 2717.57 ± 769.10 a | 1199.96 ± 72.24 b | 643.75 ± 117.22 c | 750.21 ± 52.87 bc | 9.351 | 0.0054 |
|  | Total Ni (mg·kg^-1^) | 61.86 ± 6.49 b | 96.69 ± 18.05 ab | 157.64 ± 31.36 a | 157.64 ± 31.45 a | 3.641 | 0.0639 |
|  | Total Pb (mg·kg^-1^) | 6486.27 ± 1574.78 b | 8616.89 ± 268.49 b | 10556.31 ± 2574.02 b | 27288.66 ± 356.09 a | 7.845 | 0.0091 |
|  | Total Zn (mg·kg^-1^) | 6264.83 ± 200.29 b | 6361.72 ± 738.85 b | 10473.87 ± 2094.27 b | 36940.23 ± 1911.73 a | 28.6 | < 0.001 |
|  | Total Sn (mg·kg^-1^) | 1884.44 ± 251.82 a | 1625.56 ± 255.42 a | 1035.56 ± 276.99 ab | 643.33 ± 54.64 b | 5.028 | 0.0302 |
| Available metal | Available Cd (mg·kg^-1^) | 1.66 ± 0.42 b | 2.79 ± 1.11 b | 11.52 ± 2.58 a | 1.65 ± 0.21 b | 7.316 | 0.0111 |
|  | Available Cu (mg·kg^-1^) | 44.41 ± 11.65 a | 13.57 ± 3.66 b | 7.46 ± 2.37 bc | 3.63 ± 0.43 c | 10.76 | 0.00351 |
|  | Available Pb (mg·kg^-1^) | 190.92 ± 52.95 b | 190.38 ± 34.84 ab | 170.77 ± 39.22 b | 468.87 ± 30.61 a | 3.07 | 0.0909 |
|  | Available Cr (mg·kg^-1^) | 0.42 ± 0.04 a | 0.12 ± 0.05 b | 0.06 ± 0.01 b | 0.16 ± 0.04 b | 9.67 | 0.00489 |
| Physical and ionic properties | Temperature (℃) | 14.80 ± 0.53 ab | 12.37 ± 0.32 b | 12.73 ± 0.73 ab | 16.47 ± 1.82 a | 2.685 | 0.117 |
|  | ORP (mV) | 191.33 ± 28.58 b | 214.67 ± 15.96 ab | 292.33 ± 9.68 a | 239.33 ± 28.88 ab | 2.296 | 0.155 |
|  | MC (%) | 8.69 ± 1.17 b | 15.84 ± 2.88 b | 28.22 ± 1.178 a | 13.78 ± 2.82 b | 6.689 | 0.0143 |
|  | pH | 8.68 ± 0.02 ab | 8.74 ± 0.17 ab | 8.46 ± 0.08 b | 8.91 ± 0.04 a | 2.604 | 0.124 |
|  | EC (μS/cm) | 115.37 ± 12.00 a | 99.77 ± 14.80 a | 110.97 ± 10.76 a | 87.93 ± 3.14 a | 0.764 | 0.545 |
| Nutritive elements and carbon | Total K (mg·kg^-1^) | 1861.67 ± 967.79 a | 840.00 ± 237.63 a | 884.00 ± 192.13 a | 1025.33 ± 91.05 a | 0.221 | 0.879 |
|  | Total P (mg·kg^-1^) | 3.58 ± 0.54 a | 4.18 ± 0.29 a | 4.13 ± 0.27 a | 4.34 ± 0.19 a | 0.745 | 0.555 |
|  | Total C (%) | 4.74 ± 1.19 a | 4.50 ± 1.06 a | 2.60 ± 0.65 a | 2.82 ± 0.46 a | 0.796 | 0.53 |
|  | Total N (%) | 0.07 ± 0.02 b | 0.15 ± 0.03 b | 0.32 ± 0.04 a | 0.16 ± 0.05 b | 5.606 | 0.0229 |
|  | C: N | 92.61 ± 33.62 a | 32.21 ± 8.32 ab | 7.70 ± 1.38 b | 25.32 ± 9.91 ab | 3.393 | 0.0742 |
| ORP, oxidation reduction potential; MC, moisture content; EC, electrical conductivity | | | | | |  |  |


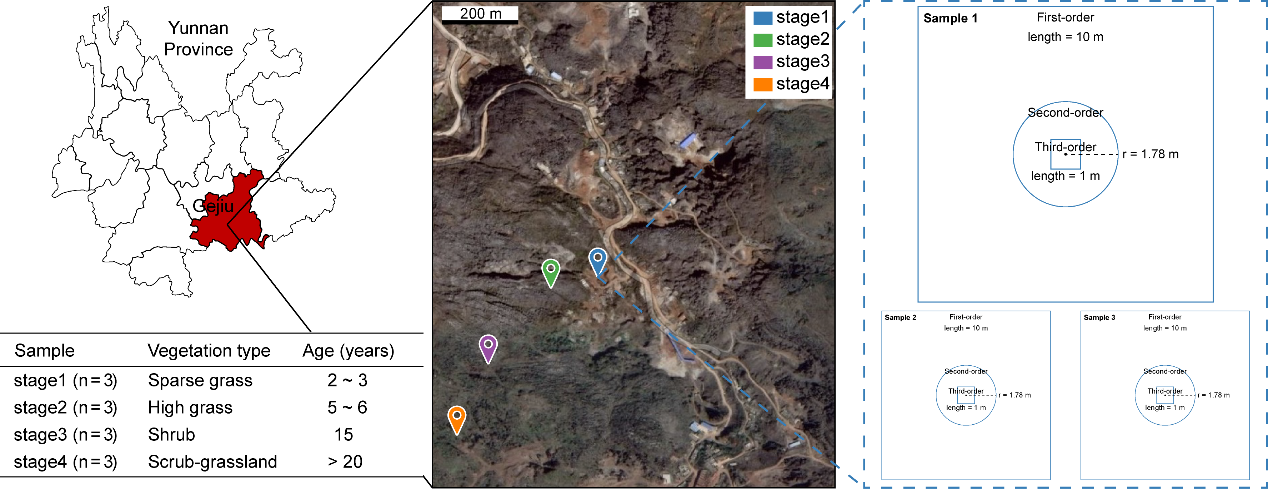


Figure S1 Study area and the distribution and description of sampling site, spatial scales of plots


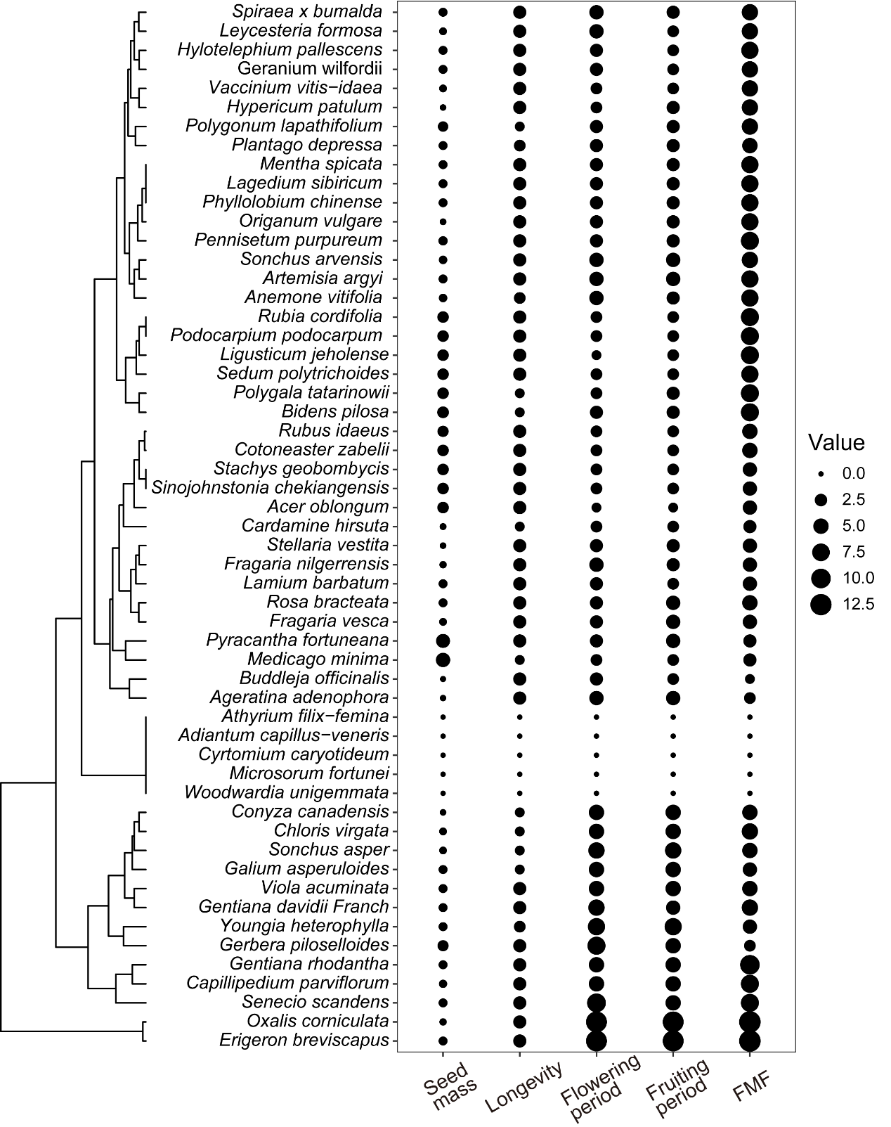


Figure S2 Distribution of the 5 regenerative traits across the phylogeny of the all species (*n* = 55 species). The cluster tree is calculated from five regeneration traits of all species occurred in four successional stages. The size of black solid dots represents the value of each regenerative traits. FMF is first month of flowering. The longevity value ranges from 1 to 4, the flowering period is between 0 and 12, the fruiting period is between 0 and 12, the value of FMF is between 0 and 13, and the seed mass is between 0 and 4 g.


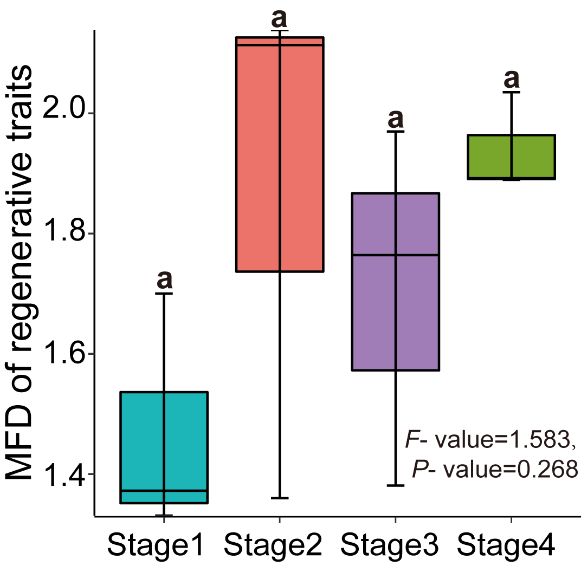


Figure S3 Mean pairwise functional distance (MFD) of comprehensive regenerative traits in different successional stages (*n* = 12 plots). Different letters indicate significant differences between stages based on 95% confidence intervals (*P* < 0.05) obtained using ANOVA and the LSD test. Stage 1 = 2-3 years, Stage 2 = 5-6 years, Stage 3 = 15 years, Stage 4 > 20 years.

Before mantel test, we used the ‘varclus’ procedure in the ‘Hmisc’ R package to assess the collinearity or redundancy of environmental factors (Jiarpakdee et al. 2016), some clustering trees were constructed and the threshold equal to or less than 0.7 was chose to delete highly redundant factors (Wang et al. 2017; Wu et al. 2021).


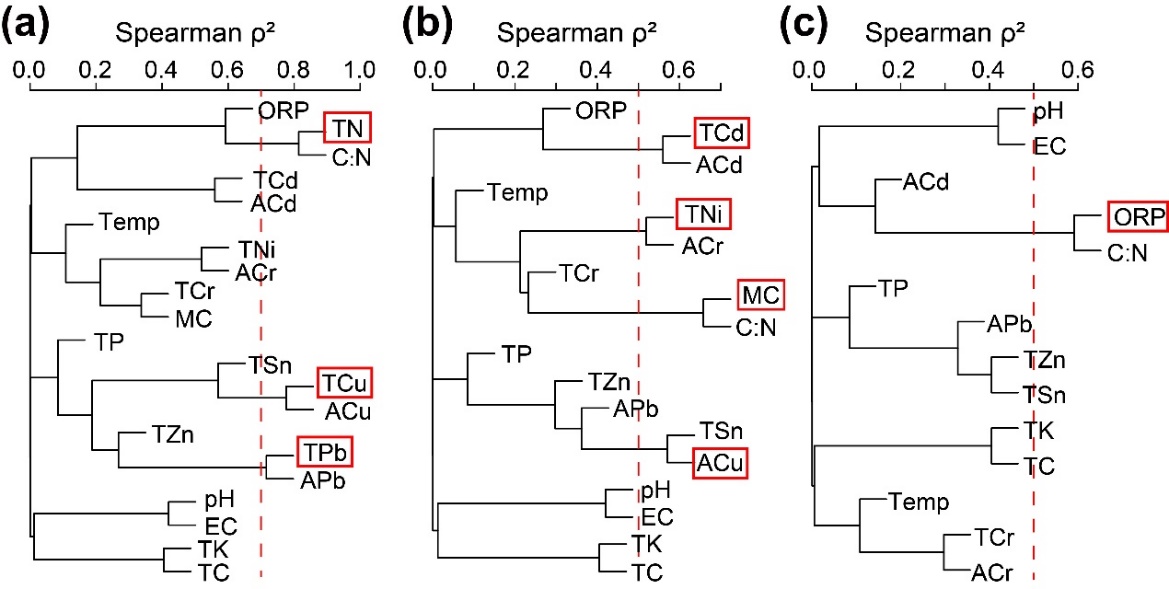


Fig S4 Cluster analysis of soil conditions: (a) Initial screening with the criterion of a Spearman's ρ^2^ > 0.7; (b) Second screening step with the criterion of a Spearman's ρ^2^ > 0.5; (c) Third screening step with the criterion of a Spearman's ρ^2^ > 0.5. Spearman distance was used for clustering, and environmental factors from branches exceeding the screening criteria were removed. The red dotted line represents the clustering coefficient criteria, and the red box represents the eliminated environmental conditions.


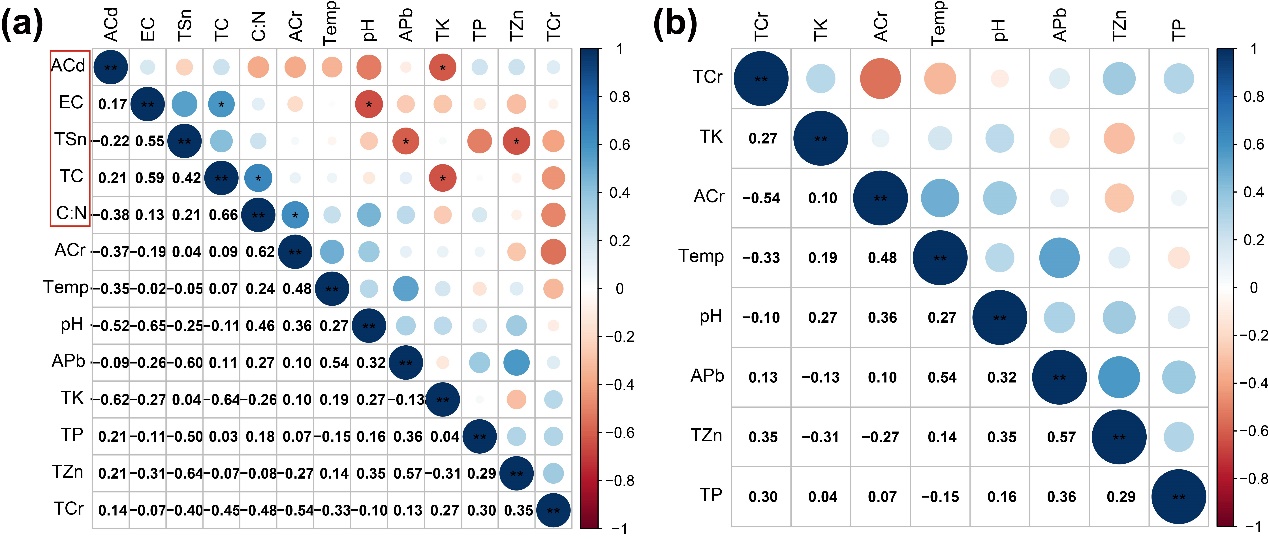


Figure S5 Correlation heat map according to environmental conditions: (a) Environmental factors retained after cluster analysis; the red box represents eliminated environmental conditions with significant correlations. (b) Correlations between the factors remaining after those with significant correlation were removed.

**Reference**

Jiarpakdee, J., Tantithamthavorn, C., Ihara, A. & Matsumoto, K. (2016) A Study of Redundant Metrics in Defect Prediction Datasets. 51-52.

Wang, X.-B., Lü, X.-T., Yao, J., Wang, Z.-W., Deng, Y., Cheng, W.-X., Zhou, J.-Z. & Han, X.-G. (2017) Habitat-specific patterns and drivers of bacterial β-diversity in China’s drylands. The ISME journal, 11, 1345-1358.

Wu, M.-H., Chen, S.-Y., Chen, J.-W., Xue, K., Chen, S.-L., Wang, X.-M., Chen, T., Kang, S.-C., Rui, J.-P. & Thies, J.E. (2021) Reduced microbial stability in the active layer is associated with carbon loss under alpine permafrost degradation. Proceedings of the National Academy of Sciences, 118.
